# Removal of biogenic amines from wines by chemisorption on functionalized silica and effects on other wine components

**DOI:** 10.1038/s41598-020-74287-3

**Published:** 2020-10-14

**Authors:** Juan José Rodríguez-Bencomo, Peggy Rigou, Fulvio Mattivi, Francisco López, Ahmad Mehdi

**Affiliations:** 1 ICGM, Univ. Montpellier, CNRS, ENSCM, Montpellier, France; 2 UMR 1083 Sciences Pour L’Œnologie, INRA, Montpellier SupAgro, Univ. Montpellier, Montpellier, France; 3 University of Trento, Department of Cellular, Computational and Integrative Biology – CIBIO, San Michele all’Adige, Italy; 4 Department d’Enginyeria Química, Facultat d’Enologia, Universitat Rovira i Virgili, Tarragona, Spain; 5 Present Address: Agrotecnio - Centre for Food and Agriculture Research, Av. Rovira Roure 191, 25198 Lleida, Spain

**Keywords:** Analytical chemistry, Biochemistry, Chemical safety

## Abstract

The effectiveness of several functionalized silica materials (cation-exchange materials) for the removal of biogenic amines from wines, and the effects on other wine components and organoleptic characteristics were evaluated. Results have shown that mesoporous silica material bi-functionalized with phosphonic and sulfonic acids allowed the removal of histamine, putrescine, cadaverine, spermine and spermidine from wines, although the dose must be adapted for each wine according to the removal requirements and wine characteristics. A plus of the adsorbent developed is that it can be recovered and re-used for at least 3 treatments. Immediately following the treatments, a decrease in the levels of linear ethyl esters (ethyl hexanoate, ethyl octanoate and ethyl decanoate) was observed, although these levels were re-equilibrated after several days reducing this undesired side effect. A slight, but perceptible, effect on wine color was observed, probably due to the slight decrease in the pH of the wine produced by the treatments. On the basis of the sensory analysis that focused only on the aroma of the wines, the proposed technique would be more adequate for wines aged in barrels than for young wines.

## Introduction

The presence of biogenic amines (BA), such as histamine, putrescine and cadaverine, is very common in fermented foods due mainly to microorganism metabolism^1^. In the case of wines, the presence of histamine, in particular, is beginning to generate concern due to its negative effects on health, which are enhanced by the presence of alcohol^2,3^. Although the International Organization of Vine and Wine (OIV) has not indicated any maximum level for BA, it has recommended minimizing its presence in wines^4^. However, some countries have implemented regulations based in maximum recommended levels for some BA^5,6^.

Although BA can be present in grapes, they are mainly produced during winemaking, most critically during malolactic fermentation (MLF). MLF is a second fermentation carried out by lactic acid bacteria (LAB) after alcoholic fermentation, such fermentation being more common in red wine than in white wines^7^. Along these lines, several previous studies focused on the selection of LAB starters with low BA production. Moreno-Arribas et al.^8^ concluded that the capacity of the LAB to produce BA could be more related to the strain than to the specific species of microorganism. However, Landete et al.^9^ reported a very high BA production trend for some species. Morevorer, Landete et al.^10^ evaluated the production of BA by several LAB in synthetic and real wines. They observed that the main origin of histamine, tyramine and 2-phenylkethylamine were certain LAB strains, but levels of putrescine, cadaverine and tryptamine could not be related to the action of microorganisms in winemaking. More recently, Berbegal et al.^11^ selected a starter from the indigenous LAB of the vineyard and obtained up to 5 times less histamine in an industrial Tempranillo wine inoculated with the selected strain compared to spontaneous malolactic fermentation. At first glance, these results are interesting, however it is well known that the production of BA is strongly dependent on the viticultural and enological practices that can affect the grape and must composition, and more particularly, the composition of amino acids^5,7^.

Cueva et al.^12^ evaluated degradation of BA by microorganisms during winemaking. They reported degradation of histamine, tyramine and putrescine by different fungi isolated from grapevines and vineyard soil. The application of selected fungi to wines showed better histamine degradation for red wines than for white wines. García-Ruiz et al.^13^ have also evaluated the capacity of some LAB to degrade BA, however, the effectiveness observed in synthetic media were strongly limited by the wine matrix. The main drawback of the inoculation of musts and wines with microorganisms that are different to those normally used in winemaking is the strong risk of modification of the organoleptic characteristics and of the quality of the final wines, as a result of enzymatic reactions and of the metabolism of the microorganism.

As an alternative to the previous approaches based on production mitigation and degradation of the BA, other studies have focused on the removal of the BA from the final wines. The studies carried out under usual enological treatments, clarification and fining, have shown that the most effective coadjuvant was bentonite^14,15^. However, although bentonite is currently used in white winemaking for protein removal in order to avoid wine turbidity after bottling, the drawbacks of its use, related to the removal of volatile compounds, thus affecting aroma, are also well known^16,17^. Therefore, this approach for the BA removal by using adsorbents should be addressed searching for the selectivity on the removal process.

So far, research aimed at finding specific adsorbents for BA removal from wines remains very limited. Amghouz et al.^18^ assayed a cation exchange adsorbent (a modified zirconium phosphate particles) in synthetic wines based on the most logical retention mechanism of BA, related to the protonated state of the amines at the wine pH. However, only one publication addresses the study of the effect of the structure and type of cation exchange functionalization of the adsorbent on the retention of BA in synthetic wines^19^. In that study, the authors investigated several silica structures and cation exchange functionalizations and concluded that macroporous and mesoporous (SBA-15 type) structures functionalized with phosphonic and sulfonic acids allowed an effective retention and removal of five BA (histamine, spermine, spermidine, putrescine and cadaverine). In addition, they obtained high removal rates with a lamellar material functionalized only with sulfonic acid. They also reported that the effectiveness of the removal method strongly depends on the number of amine functions on the BA molecule; the method being much more effective (close to 100%) for spermine and spermidine removal than for monoamines such as isoamylamine, tyramine and 2-phenylethylamine. These observations were attributed to the multiple interaction points occurring with the surface of the adsorbent when more than one amine group is present in the BA, thus stabilizing the retention on the solid. Starting from that previous study, the present study hypothesizes that a controlled structure of the adsorbents with an adequate functionalization would allow a selective retention of the BA in real wines, minimizing the risks of loss of other wine components that could affect wine quality. Therefore, the aim of this work was to evaluate the effectiveness of these adsorbents previously designed^19^ for the removal of biogenic amines from real wines and also evaluating the effect on other wine components and on its sensorial quality. In addition, the practical aspects of the application of the adsorbents to real wines were also studied.

## Results and discussion

### Removal of biogenic amines from white and red wines

On the basis of the previous results obtained in synthetic wine media^19^, a lamellar material functionalized with sulfonic acid groups and macroporous and mesoroporous (SBA-15 type) xerogel structures bi-functionalized with phosphonic and sulfonic acids were selected to evaluate their effectiveness in real wine samples. For this purpose, a young red wine and a white wine fortified with the eight BA studied: histamine, putrescine, cadaverine, spermine, spermidine, isoamylamine, 2-phenylethylamine and tyramine were treated with the three solids (dose of solid 5 g/L). The chemical formula, molecular weight and molecule length of each biogenic amine are detailed in Supplementary Table S1.

**Figure 1:**
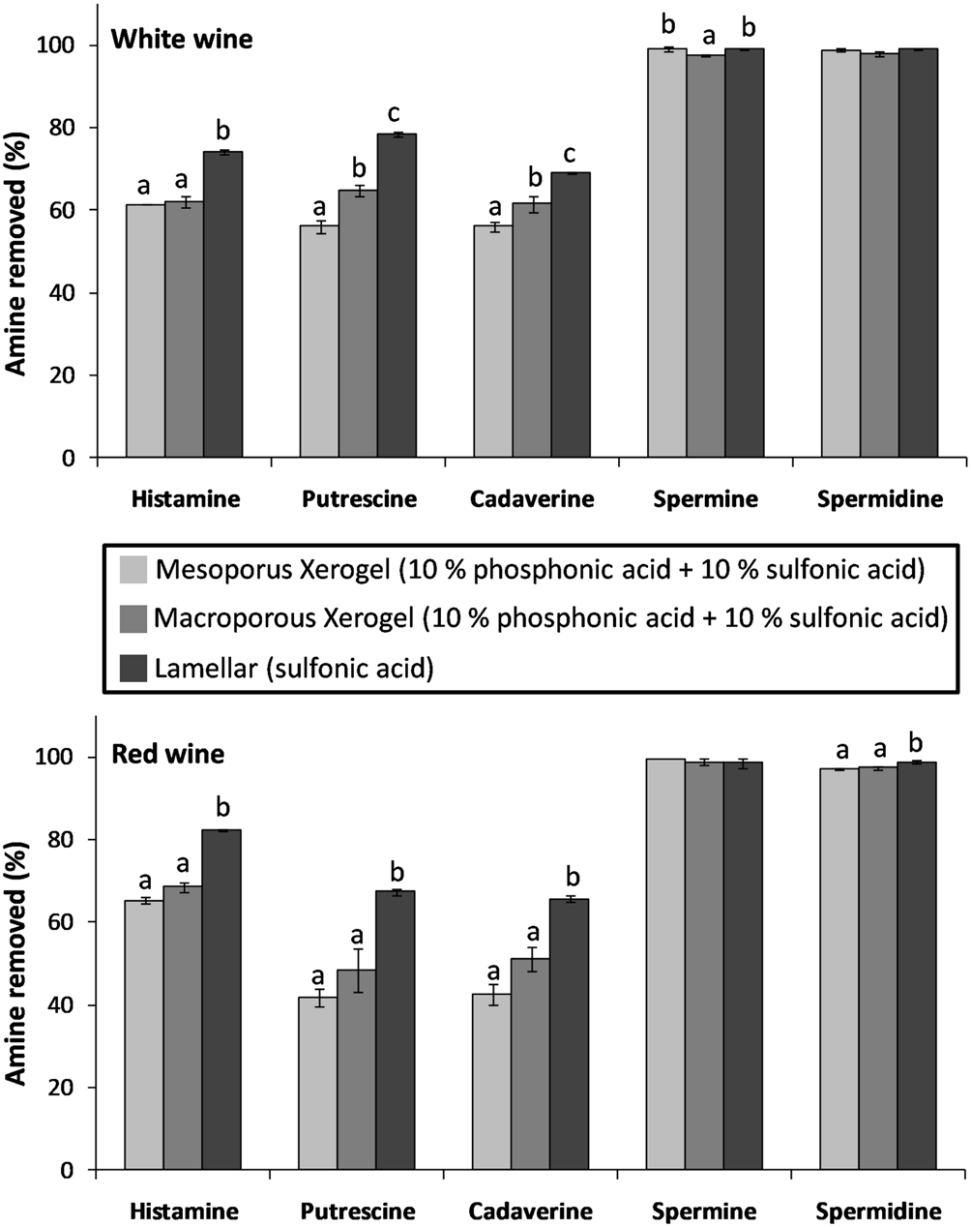
Biogenic amine removal (%) from white and red wines with macroporous xerogel (10% of phosphonic acid + 10% of sulfonic acid), mesoporous xerogel (10% of phosphonic acid + 10% of sulfonic acid) and sulfonic acid functionalized lamellar materials (solid dose used 5 g/L). Different letters indicate statistics differences in ANOVA (p < 0.05) and the LSD test.

As expected, the removal of the three monoamines, isoamylamine, 2-phenylethylamine and tyramine, was limited, especially when using both xerogels (data not shown). For the lamellar sulfonic acid material, the removal rate was around 25% and 17% for 2-phenylethylamine and tyramine, respectively, and practically null for isoamylamine. These very low removal percentages can be attributable to the complex chemical composition of the wine matrix and the competitive effects of molecules for the active sites on the solid, being more important in the case of amine molecules with only one amine group and therefore with a weak strength of interaction with the solid^18^. These results obtained when using a high solid dose for the treatment of wines (5 g/L) clearly show the limitation of these materials for the removal of monoamines. Consequently, we decided to focus further work on the other five BA only. Figure 1 shows the removed percentage of those five BA (histamine, cadaverine, putrescine, spermine and spermidine) from red and white wines with the three types of materials assayed. As can be seen, for compounds with more than two amine groups such as spermine and spermidine, all three materials presented a very high level of effectiveness (> 97%) at a solid dose of 5 g/L for both types of wine. These results reinforce the idea that multiple points of interaction of the amine molecule with the surface of the solid stabilize retention, avoiding displacement by other wine components^18^. On the other hand, histamine, cadaverine and putrescine presented statistical differences in removal percentages due to the type of material in the two types of wines. In all cases, the lamellar material showed the best removal capacity. The macroporous xerogel presented slightly higher removal capacity than the mesoporous xerogel, though the difference was significant for white wine only. In addition, the removal percentages tended to be generally higher for histamine than for the linear diamines (cadaverine and putrescine). Considering the effect of the type of wine, for histamine, removal efficiency was higher for red wine (65.3–82.2%) than for white wine (61.4–74.1%). However, the effect of the type of wine was the opposite for cadaverine and putrescine (p value < 0.01). When comparing those results with the assays performed in wine model solutions^19^, we observe that the removal percentages were lower for the real wines, especially in the case of both xerogels (with effectiveness losses of up to 50%). Those differences in removal efficiency show the important influence of the wine matrix and most particularly the wine components such as proteins, polyphenols, sugars, etc., that can interact with the active sites of materials and could also block the pores of the silica structure. Taking into account that red wines present a much more complex matrix than white wines, the different behaviors observed for these three compounds (histamine, cadaverine and putrescine), where linear amines are more affected by the matrix than histamine, can be also related to structural aspects of these amine molecules such as distance between functional groups and the rigidity of the molecule^18,20^.

### Effects of the adsorbent treatment on other wine components and parameters

In order to evaluate the effect of the treatment with the materials on other wine components and wine parameters, control wines and treated wines (with a dose of adsorbent of 5 g/L) were analyzed. Table 1 shows the total protein and aminoacid content, color parameters (color intensity and tonality) and pH for the control and treated red and white wines. A significant decrease in pH (between 0.13 and 0.29 units of pH) was observed after the treatment with all the solids and for both types of wine. This effect was greater for the lamellar material and is attributed to the higher ratio of acids groups compared to the mesoporous and macroporous xerogels materials. Although this decrease of pH could be avoided by using the solids as potassium salts, it could also be interesting for improving the microbiological stability for wines with high pH^21,22^.

**Table 1:** General composition and parameters of control and treated wines.

	Control	Mesoporous X	Macroporous X	Lamellar
**White Wine**^**a**^				
pH	3.53 ± 0.00	3.40 ± 0.001*	3.39 ± 0.00*	3.24 ± 0.00*
Aminoacids(mg/L)	52.3 ± 2.7	52.8 ± 4.9	52.1 ± 0.1	48.9 ± 4.1
Proteins (mg/L)	12.8 ± 1.5	6.90 ± 0.26*	6.39 ± 0.43*	3.59 ± 0.58*
Color intensity	0.109 ± 0.003	0.104 ± 0.002	0.106 ± 0.001	0.112 ± 0.003
Tonality	4.714 ± 0.142	4.377 ± 0.082	4.283 ± 0.202	4.050 ± 0.045*
Total polyphenol index	9.01 ± 0.14	9.26 ± 0.40	9.07 ± 0.27	8.63 ± 0.13
**Red wine**^**a**^				
pH	3.62 ± 0.01	3.42 ± 0.00*	3.42 ± 0.00*	3.34 ± 0.01*
Aminoacids (mg/L)	53.3 ± 2.0	53.4 ± 0.1	53.7 ± 1.3	48.2 ± 1.2
Proteins (mg/L)	145 ± 4	137 ± 4	141 ± 4	128 ± 1*
Color intensity	10.485 ± 0.025	10.366 ± 0.008	10.255 ± 0.026	9.644 ± 0.060*
Tonality	0.798 ± 0.001	0.764 ± 0.001*	0.763 ± 0.001*	0.758 ± 0.001*
Total polyphenol Index	55.81 ± 1.22	53.55 ± 0.02	52.44 ± 0.85	50.9 ± 2.45

*Indicate Statistical differences in ANOVA (p < 0.05) and Dunnett test (respect to control).

^a^Commercial young wines (dry wines; Alcohol = 12% v/v).

The total protein content of red wines only showed statistical differences in the case of lamellar material (a decrease of 12%). In the case of white wine, the levels of proteins were much lower than in red wine, probably because white wine was previously treated with bentonite. However, the percentage decrease of proteins observed for white wines was very high (≥ 50%) for the three materials assayed (especially with the lamellar material). This high removal effect of proteins by the lamellar material is probably related to the higher number of active cation exchange sites of the material, but also to its structure, which is similar to bentonite^23^. The minor removal effect of both xerogels (macroporous and mesoporous) could be related to their porous structure, which could limit access to the active sites by macromolecules such as proteins. In the end, none of these three materials produced a significant decrease of the total content of amino acids, which is consistent with the weak interaction of monoamines observed.

Regarding the chromatic parameters, tonality presented statistical differences for all treated red wines, whilst color intensity showed differences only for red wines treated with the lamellar material. Regarding white wine, we only observed a significant effect on the tonality for wines treated with the lamellar material. Taking into account that the total polyphenol index did not show statistical differences, the slight decrease observed in the chromatic parameters could be related to the pH variation induced by the treatments^24^. However, after treatment of red wines (and washing process of the adsorbent materials), the solids remained slightly colored (Supplementary Fig. S1), suggesting that slight adsorption of phenolic compounds had occurred.

Another important aspect to evaluate is the impact on the volatile composition responsible for the aroma of wines. As was already reported for the use of bentonite as a fining agent in winemaking, the removal of volatile compounds is a critical aspect that could limit the maximum dose used^16^. To get a better insight into the removal of aroma compounds due to contact with the materials, the volatile compounds of control and treated wines were extracted with dichloromethane just after treatment with the materials and analyzed by gas chromatography (GC). Table 2 reports the composition in aroma compounds such as alcohols, esters and acids for treated white and red wines, expressed as percentage deviation with respect to the control wine (0% indicate the same concentration as the control wine). To evaluate the effects, in addition to the ANOVA analysis and a Dunnett test, the criterion of a deviation higher than ± 15% (from the control wine) was applied to consider that the samples were sufficiently different to produce an effect on the aroma quality. Results show that alcohols were not affected by any of the treatments. Regarding the organic acids chemical group, the most intense removal effects were observed for decanoic acid (between 19.9 and 14.2%) in red wines and dodecanoic acid (between 61.8 and 40.3%) in white wines. Concerning the ester chemical group, ethyl ester content of hexanoic, octanoic and decanoic acids showed a strong decrease in all treated wines (red and white), such decrease affecting most specifically the ethyl octanoate and ethyl decanoate (decrease higher than 75%). Among esters of acetate, only hexyl acetate in white wines showed a decrease in concentration, of around 30–35%. This strong reduction in the content of these esters will affect the fruity aroma since these compounds are responsible (at least in part) for the aromatic character^25^. However, the concentration of esters is mainly regulated by the chemical equilibrium with alcohols (ethanol in the case of ethyl esters) and acids. Therefore, we may predict a re-equilibrium of the contents over time, especially for the most affected esters and acids^25,26^. Considering that our materials are functionalized with cation exchange groups, interaction of esters and acids by these mechanisms is not likely to occur given the pH of the wine. However, a retention effect due to hydrogen bonding with silanol groups and/or an acid catalytic effect of the sulfonic groups on the hydrolysis reaction of the esters are more probable^27,28^. Therefore, a chemical and sensory evaluation (mainly focusing on aroma) of the wines after a time in storage must be carried out in order to evaluate the final effect of the treatments.

**Table 2:** Volatile composition (expressed as percentage deviation respect to control wines) for the samples treated with the silica materials.

	White Wine			Red Wine		
	Mesoporous X	Macroporous X	Lamellar	Mesoporous X	Macroporous X	Lamellar
**Alcohols**						
Propanol	3.2 ± 6.8	− 2.4 ± 1.7	12.6 ± 1.3	− 0.7 ± 0.6	0.9 ± 4.4	− 1.4 ± 3
Isobutanol	2.3 ± 0.1	2.8 ± 0.7	6.6 ± 1.4	2.1 ± 0.4	4.6 ± 3.4	4.4 ± 0.9
2-Methyl-1-butanol	8.3 ± 3.3	1.1 ± 3.4	9.1 ± 2.2	− 4.2 ± 1.1	− 1.5 ± 0.4	− 3.4 ± 0.8
3-Methyl-1-butanol	9.6 ± 2.7	1.8 ± 3.9	8.5 ± 2.2	− 4.3 ± 0.4	− 2.0 ± 2.2	− 5.5 ± 1.1
Hexanol	6.9 ± 1	− 1.7 ± 6.5	− 1.5 ± 7.9	− 5.4 ± 0.2 *	− 5.5 ± 1.2 *	− 5.0 ± 1.6 *
2-Phenylethanol	6.5 ± 1	1.5 ± 4.3	4.5 ± 3.1	− 6.6 ± 1.6 *	− 2.2 ± 2	− 6.5 ± 1.3 *
Methionol	7.2 ± 2.7	− 0.1 ± 2.4	6.9 ± 3.1	− 2.0 ± 1.0	− 2.8 ± 2.5	1.1 ± 0.7
**Esters**						
Ethyl propanoate	8.1 ± 10.8	− 2.6 ± 3.4	− 7.5 ± 10.3	− 10.3 ± 0.4	− 11.8 ± 0.9	− 2.2 ± 7.5
Ethyl isobutyrate	5.6 ± 8.5	− 7 ± 3.8	− 9.6 ± 8.8	− 10.1 ± 0.6 *	− 13.9 ± 1.7 *	− 3.9 ± 4.7
Ethyl butanoate	− 0.8 ± 0.6	− 7.4 ± 4.1	− 7.6 ± 5.9	− 9.3 ± 1 *	− 9.6 ± 2.3 *	− 6.9 ± 1.4 *
Ethyl 2-methylbutyrate	− 3.4 ± 0.4	− 8.6 ± 3.5	− 9 ± 5.1	− 12 ± 0.2	− 12.4 ± 2.4	− 9.3 ± 1.7
Ethyl isovalerate	− 4.0 ± 3.6	− 3.3 ± 2.4	− 12 ± 4.6	− 14.2 ± 0.6 *	− 13.9 ± 1.7 *	− 11.5 ± 1.9 *
Ethyl hexanoate	− 31.8 ± 0 *	− 37.6 ± 2.6 *	− 36.4 ± 1.7 *	− 31.1 ± 0.4 *	− 31.8 ± 0.5 *	− 29.6 ± 0.7 *
Ethyl octanoate	− 84.6 ± 0.3 *	− 86.6 ± 0.4 *	− 86.9 ± 0.8 *	− 80.6 ± 0.2 *	− 81.1 ± 0.6 *	− 79.3 ± 0.7 *
Ethyl decanoate	− 88.5 ± 0.5 *	− 89.4 ± 0.5 *	− 97.9 ± 0.1 *	− 92.2 ± 1.3 *	− 92.3 ± 0.9 *	− 94.0 ± 0.1 *
Ethyl dodecanoate	− 0.5 ± 0.4	− 2.1 ± 0.7	4.9 ± 2 *	− 5.9 ± 0.7	0.9 ± 3.3	− 5.1 ± 1.2
Ethyl lactate	− 0.7 ± 4.8	− 2.1 ± 1.3	− 0.8 ± 3.6	0.9 ± 2.3	1.9 ± 3.1	1.5 ± 1.8
Diethyl succinate	3.9 ± 0.3	− 3.0 ± 3.2	6.8 ± 3.2	− 4.6 ± 1.9	− 0.8 ± 0.7	− 4.3 ± 0.8
Propyl acetate	6.2 ± 0.9	− 2.8 ± 3.3	− 4.4 ± 4.7	− 8.3 ± 0.2 *	− 9.8 ± 1.6 *	− 5.2 ± 2.2
Isobutyl acetate	3.6 ± 1.6	− 2.3 ± 3.2	− 4.3 ± 6.8	− 9.4 ± 0.4 *	− 8.6 ± 1.9 *	− 8.8 ± 0.8 *
Isoamyl acetate	− 5.1 ± 2.1	− 8.9 ± 5	− 11.9 ± 5.4	− 12.3 ± 0.9 *	− 12.7 ± 1.5 *	− 10.1 ± 2.1 *
2-Methylbutyl acetate	− 4.3 ± 2.3	− 8.3 ± 5.2	− 11.7 ± 4.9	− 13.1 ± 0.4 *	− 12.7 ± 1.2 *	− 11.4 ± 1.1 *
Hexyl acetate	− 29.6 ± 0.3 *	− 35.4 ± 2.4 *	− 34.8 ± 2.1 *	d-n.q	d-n.q	d-n.q
2-phenylethyl acetate	− 0.3 ± 1.5	− 6.3 ± 3.4	− 0.8 ± 3.3	− 1.6 ± 0.6	− 2.8 ± 0.1	1.6 ± 4.5
**Acids**						
Propanoic acid	13.4 ± 6.2	2.3 ± 3.2	9.5 ± 3.4	− 5.1 ± 2.7	− 4.4 ± 1.2	5.1 ± 5.1
Butanoic acid	11.5 ± 0.4	3.4 ± 1.3	10.4 ± 7.3	− 4.6 ± 1.6	− 1.2 ± 0.3	5.1 ± 3.8
Hexanoic acid	11.3 ± 2.1	− 0.1 ± 3.5	12 ± 1.1 *	− 8.8 ± 1 *	− 5.8 ± 1.6	− 3.6 ± 2.3
Octanoic acid	11.4 ± 1.7	0.5 ± 5.0	9.3 ± 1.4	− 10.5 ± 1.5	− 6.3 ± 2.5	− 6.6 ± 0.5
Decanoic acid	− 1.3 ± 1.6	− 10.7 ± 4.9	− 7.1 ± 1.6	− 19.9 ± 0.6 *	− 14.2 ± 4.1 *	− 18.6 ± 2 *
Dodecanoic acid	− 56.7 ± 1.7 *	− 61.8 ± 0.6 *	− 40.3 ± 6.5 *	− 12.1 ± 6.4	− 2 ± 1.3	− 7.2 ± 5.8

*d-n.q* detected-not quantified.

*Indicate statistical differences in ANOVA (p < 0.05) and in Dunnet test respect to the control wines.

The impact of the lamellar material observed on pH, protein content and chromatic parameters of the wines probably makes this material the most problematic for the organoleptic characteristics of wines. Although the behaviors of both xerogels were generally similar, a slightly lower effect of mesoporous xerogels was observed in regard to chromatic parameters in red wines. The subsequent experiments of this study were focused only on red wines, since these wines more commonly present high levels of BA, and on their treatment for BA removal by the mesoporous xerogel material.

### Effect of red wine type and dose of solid on biogenic amine removal

Previous results have shown the effect of treatments with the materials on some wine parameters and components using a dose of 5 g/L. However, at a practical level, the removal of BA should be carried out taking into account the legal or recommended limit for each amine, and the need to minimize side effects, so in most real cases the optimal dose will probably be lower than those used in previous experiments. Thus, different doses (from 1 to 5 g/L) of the selected material (mesoporous xerogel) were evaluated in three different red wines. Table 3 presents, for each wine, the retention percentage for each amine at each dose of material assayed (wine characteristics are also detailed in this table). Results were treated with a two-way ANOVA analysis (dose of solid and type of wine) that is also reported in Table 3. As expected, a clear effect of the dose of solid was observed on the efficiency of amine removal, except for spermine that presented removal percentages higher than 92.4% at all doses. This high retention of spermine (even at low dose of solid) is related to the number of amine functions (4 in this molecule) that allows a very stable retention and thus avoids competitive effects for the active sites of the solid. For the other BA (histamine, cadaverine, spermidine and putrescine), the general trend observed on amine removal is almost linear with the dose used. For a dose of solid of 1 g/L, spermidine showed removal percentages higher than 63%. However, the removal effectiveness for histamine, cadaverine and putrescine was very limited (< 28.1%) with this low dose of solid.

**Table 3:** Biogenic amine removal (expressed as %) for three different red wines treated with four different doses of adsorbent silica material.

	1 g/L	1.5 g/L	2.5 g/L	5 g/L
**Red wine vintage 2016. Grape variety Lagrein. TPI = 70.3. Ethanol = 13.0%**				
Histamine	14.5 ± 0.6	26.2 ± 0.3	35.9 ± 7.3	55.8 ± 4.3
Cadaverine	9.3 ± 3.0	18.4 ± 0.2	29.2 ± 1.4	43.5 ± 2.2
Putrescine	< 5%	11.8 ± 1.3	28.5 ± 4.4	39.7 ± 1.8
Spermine	97.2 ± 0.6	98.8 ± 0.1	93.5 ± 8.2	99.7 ± 0.1
Spermidine	63.1 ± 2.3	80.1 ± 0.3	88.0 ± 0.6	94.3 ± 0.4
**Red wine vintage 2013. Grape variety: Tempranillo. TPI = 50.3. Ethanol = 13.5%**				
Histamine	15.1 ± 6.7	29.3 ± 4.8	36.2 ± 4	58.6 ± 1.5
Cadaverine	14.8 ± 0.3	22.9 ± 8.5	32.7 ± 1.0	45.6 ± 0.7
Putrescine	< 5%	16.6 ± 9.9	23.9 ± 1.0	32.9 ± 1.4
Spermine	97.2 ± 0.2	98.2 ± 0.2	98.7 ± 0.2	99.0 ± 0.8
Spermidine	66.4 ± 2.8	78.6 ± 1.4	87.3 ± 0.1	94.7 ± 1.1
**Red wine vintage 2016. Grape variety: Pinot Noir. TPI = 58.3. Ethanol = 14.0%**				
Histamine	28.1 ± 1.1	32.3 ± 1.8	42.3 ± 3.2	58.5 ± 2.9
Cadaverine	13.1 ± 0.5	17.7 ± 1.3	23.9 ± 0.5	39.4 ± 3.5
Putrescine	7.51 ± 3.66	8.78 ± 9.03	16.7 ± 3.8	35.7 ± 1.9
Spermine	92.4 ± 1.7	94.2 ± 1.2	94.4 ± 0.7	92.6 ± 0.5
Spermidine	69.0 ± 1.5	79.7 ± 1.5	87.4 ± 1.3	94.3 ± 0.3

Two Way-ANOVA (wine type and solid dose) results (p < 0.05):

Wine effect significant for Histamine, Cadaverine and Spermine.

Dose effect significant for Histamine, Cadaverive, Putrescine and Spermidine.

No significant effects for Wine-Dose interaction.

On the other hand, an effect of the wine type was observed for histamine, cadaverine and spermine removal. Histamine and spermine showed the highest and lowest average removal percentages in Pinot Noir (PN) wine respectively. However, cadaverine showed the best average removal percentages for Tempranillo (T) wine. The removal ranges observed for a fixed dose did not show great differences between wines in the case of the higher doses of solid. For example, at a dose of 5 g/L, histamine removal ranged from 55.8 to 58.6%, cadaverine ranged from 39.4% to 45.6%, and spermine from 92.4 to 97.2%. At the other end of the scale (1 g/L dose) histamine removal efficiency showed important differences, ranging from 14.5% to 28.1% among the different wines. No significant interactive effects were observed between the dose and type of wine.

As reported in Table 3, T and PN wines showed similar TPI (50.3 and 58.3, respectively), however, due to the age of wines, the phenol profile should be quite different^29,30^. Indeed, the oldest wine (T) could possibly present a higher degree of polyphenol polymerization^31^. On the other hand, Lagrein (L) wine presented highest TPI (73.3), being a wine of the same age as PN. In addition, Table S2 in supplementary data reports the levels of polyphenol compounds such as hydroxybenzoic acid derivatives, hydroxycinnamic acid derivatives and flavonoids in the control wines, emphasizing a great difference between the wines. However, on the basis of our results, the polyphenol contents cannot explain the variation of treatment efficiency from one wine to another.

Table S2 reports the effect of the treatment at the highest dose assayed (5 g/L) on the phenolic compounds content. We can observe that the wine most affected by the treatments was the oldest one (T). In this wine, myricetin and laricitrin content showed a significant decrease (> 28.3%). Caftaric, p-coumaric and fertaric acid content also presented a significant decrease percentage from 15.1 and 20.8%. However, epigallocatechin showed a 27.6% increase of content with respect to the control wine. On the other hand, polyphenol compounds in L and PN treated wines were much less affected by the treatment. Only myricetin showed a content decrease of 38.2% in L wine and laricitrin a 22.2% content increase in PN wine. These variations in polyphenol contents may be due to a retention effect on the solid surface or to chemical reactions occurring during the treatment and induced by the change of pH or by a catalytic effect of the adsorbent.

Therefore, considering the possible collateral effect of the treatments on wine characteristics, it may be suggested that, at a practical level, the dose of adsorbent be adapted to the type of wine, taking into account removal requirements. This should be done by preliminary assays with a view to keeping the dose to the minimum.

### Sensorial aspects: aroma and color

In order to evaluate the effect of the treatment on the organoleptic parameters (wine aroma and color), 8 different red wines were treated with a dose of 5 g/L. Treated wines were analyzed and compared with control wines (aroma sensory analysis by triangle test, color parameters and volatile composition) a week after the treatment. Results of the sensory triangle test (focused only on aroma) for each wine are presented in Table 4. As can be seen, treatment with the solids produced a significant effect (p < 0.01; according to Roessler et al.^32^) on the aroma for 6 of the wines (two young wines and four aged in barrels). Both young wines showed the highest percentage of detection by the panelists (> 63.3%), probably due to the fact that the global aroma bouquet of these wines is more dependent on esters (fermentative compounds) than the wines aged in wood that present a more complex aroma. However, wines of Lagrein and Grenache, both aged in barrels, were the wines that showed no statistical differences in triangle tests. Considering the effect of the adsorbents on some linear ethyl esters described in the previous section (mainly in the case of ethyl hexanoate, octanoate and decanoate) and the probable re-equilibration of its contents after the treatment, these esters were analyzed in control and treated samples. The contents for reference and treated wines (and the odor activity values [OAV]) of these linear esters are presented in Table 5. Generally, one week after the treatment with the solids, the concentration of these linear esters in wine decreases, but at a lower intensity when compared to concentration obtained just after the treatment (Table 3). This result can be easily explained by the re-equilibrium of the contents during the time after treatment. On the other hand, taking account the impact on the aroma of these esters (based on the OAV values), ethyl decanoate has a minimal effect on the total OAV, however, the variation of the contents of the other two compounds may strongly affect the fruity aroma perceived^25^. The decrease of the total OAV of the wines that presented statistical differences in the triangle test ranged from 8.4 to 36.4%. However, it is important to note that the two wines that did not present statistical differences showed the lowest and the highest decrease in total ester OAV, 8.3 and 51.5% for Grenache and Lagrein wines, respectively, both wines aged in barrels. This result suggests that perception of the variation in the fruity aroma of the wines could also depend on the complexity of their global aroma, and a masking effect is likely to be produced in the wines with the most complex aroma.

**Table 4:** Sensory analysis of the red wine samples treated with the solids. Triangle tests on aroma.

Wine variety (type)	Correct responses (%)
Grenache (young)	70.0 *
Tempranillo 1 (barrel)	60.7 *
Grenache (barrel)	40.0
Cabernet Sauvignon (barrel)	56.7 *
Trepat (young)	63.3 *
Tempranillo 2 (barrel)	60.0 *
Lagrein (barrel)	53.3
PinotNoir (barrel)	56.7 *

*Indicate significant differences (p < 0.01) according to Roessler et al. 1978.

**Table 5:** Contents (mg/L) [and odor activity values (OAV)] of ethyl hexanoate, ethyl octanoate and ethyl decanoate of the control and treated wines (after 1 week of the treatments with the adsorbent).

	Grenache (young)		Tempranillo 1 (barrel)	
	Control	Treated	Control	Treated
Ethyl hexanoate	0.265 ± 0.029 [18.9]	0.251 ± 0.012 [17.9]	0.412 ± 0.018 [29.4]	0.338 ± 0.002 [24.1]*
Ethyloctanoate	0.525 ± 0.069 [105]	0.341 ± 0.009 [68.3]	1.10 ± 0.05 [221]	0.913 ± 0.03 [183]*
Ethyldecanoate	0.093 ± 0.004 [0.465]	0.089 ± 0.007 [0.447]	0.215 ± 0.029 [1.07]	0.196 ± 0.013 [0.978]
Total OAV	[124]	[86.6]	[251]	[208]*

	Grenache (barrel)		Cabernet Sauvignon (barrel)	
	Control	Treated	Control	Treated
Ethylhexanoate	0.314 ± 0.023 [22.4]	0.208 ± 0.001 [14.9]*	0.304 ± 0.003 [21.7]	0.26 ± 0.021 [18.5]
Ethyloctanoate	0.408 ± 0.015 [81.7]	0.405 ± 0.009 [80.9]	0.541 ± 0.028 [108.3]	0.457 ± 0.002 [91.5]
Ethyldecanoate	0.178 ± 0.019 [0.888]	0.103 ± 0.009 [0.514]*	0.109 ± 0.004 [0.544]	0.097 ± 0.001 [0.484]*
Total OAV	[105]	[96.3]	[131]	[111]*

	Trepat (young)		Tempranillo 2 (barrel)	
	Control	Treated	Control	Treated
Ethylhexanoate	0.14 ± 0.008 [10]	0.117 ± 0.013 [8.35]	0.364 ± 0.017 [26]	0.119 ± 0.005 [8.48]*
Ethyloctanoate	0.285 ± 0.014 [57]	0.265 ± 0.001 [53]	1.073 ± 0.009 [215]	0.724 ± 0.01 [145]*
Ethyldecanoate	0.091 ± 0.006 [0.457]	0.098 ± 0.004 [0.49]	0.177 ± 0.019 [0.884]	0.144 ± 0.006 [0.72]
Total OAV	[67.5]	[61.8]	[242]	[154]*

	Lagrein (barrel)		PinotNoir (barrel)	
	Control	Treated	Control	Treated
Ethylhexanoate	0.233 ± 0.009 [16.6]	0.071 ± 0.006 [5.09]*	0.212 ± 0.017 [15.1]	0.171 ± 0.018 [12.2]
Ethyloctanoate	0.375 ± 0.006 [75]	0.197 ± 0.003 [39.3]*	0.291 ± 0.015 [58.3]	0.183 ± 0.017 [36.5]*
Ethyldecanoate	n.d	n.d	0.218 ± 0.007 [1.09]	0.225 ± 0.029 [1.12]
Total OAV	[91.6]	[44.4]*	[74.5]	[49.8]*

*n.d.* not detected.

*Indicate significant differences in ANOVA (p < 0.05). Odor activity values [OAV] are calculated by using the odor thresholds (OT) obtained from the bibliography^38^: OT (ethyl hexanoate = 0.014 mg/L; ethyl octanoate = 0.005 mg/L; ethyl decanoate = 0.2 mg/L).

Concerning the impact of treatments on wine color, CIELab parameters (Color Intensity and Tonality), the pH values for control and treated samples, and the estimation of color change (∆E_ab_), are presented in Table 6. As can be seen and as expected, the decrease in pH observed ranged from 0.13 to 0.20 units of pH, which could be the main factor responsible for the color parameter changes observed in wines^24^. Tonality in all wines showed a slight decrease, lower than 7.5%, and the color intensity only presented small variations (< 7.5%). Estimation of color change (∆E_ab_) showed values ranging from 0.794 to 7.81, and the wine with the highest color intensity was the most affected. Taking into account that variations higher than 3 for the ∆E_ab_ could be detected by the human eye^33^, most of the wines treated with the solids would suffer a slight detectable color change with respect to the control wines.

**Table 6:** The pH and color parameters (CieLab) of control and treated wines subjected to sensory analysis.

Wine	pH	Color intensity		Tonalily		∆E_ab_
	Cont-treat	Control	Treated	Control	Treated	Treat. vs Cont
Grenache (young)	3.20–3.07	14.7 ± 0.0	14.9 ± 0.0 *	1.04 ± 0.00	1.02 ± 0.00 *	5.13 ± 0.12
Tempranillo 1 (barrel)	3.48–3.30	7.89 ± 0.01	8.46 ± 0.02 *	0.842 ± 0.001	0.801 ± 0.001 *	0.794 ± 0.093
Grenache (barrel)	3.42–3.25	7.43 ± 0.03	7.32 ± 0.01 *	0.762 ± 0.003	0.744 ± 0.001 *	4.79 ± 0.11
Cabernet Sauvignon (barrel)	3.25–3.05	10.4 ± 0.0	10.5 ± 0.0 *	0.924 ± 0.001	0.909 ± 0.001 *	3.50 ± 0.00
Trepat (young)	3.19–3.04	7.30 ± 0.01	7.22 ± 0.02	0.663 ± 0.001	0.641 ± 0.000 *	4.39 ± 0.21
Tempranillo 2 (barrel)	3.67–3.49	9.74 ± 0.02	10.44 ± 0.02 *	0.930 ± 0.003	0.887 ± 0.001 *	3.58 ± 0.36
Lagrein (barrel)	3.74–3.57	22.1 ± 0.1	21.2 ± 0.0 *	1.18 ± 0.00	1.16 ± 0.00 *	7.81 ± 0.06
PinotNoir (barrel)	4.03–3.83	6.70 ± 0.04	6.70 ± 0.04	1.09 ± 0.01	1.02 ± 0.00 *	2.68 ± 0.24

*Cont* control; *Treat* treated.

*Indicate significant differences in ANOVA for color intensity and tonality (p < 0.05).

### Practical aspects for batch treatments of wines

**Figure 2:**
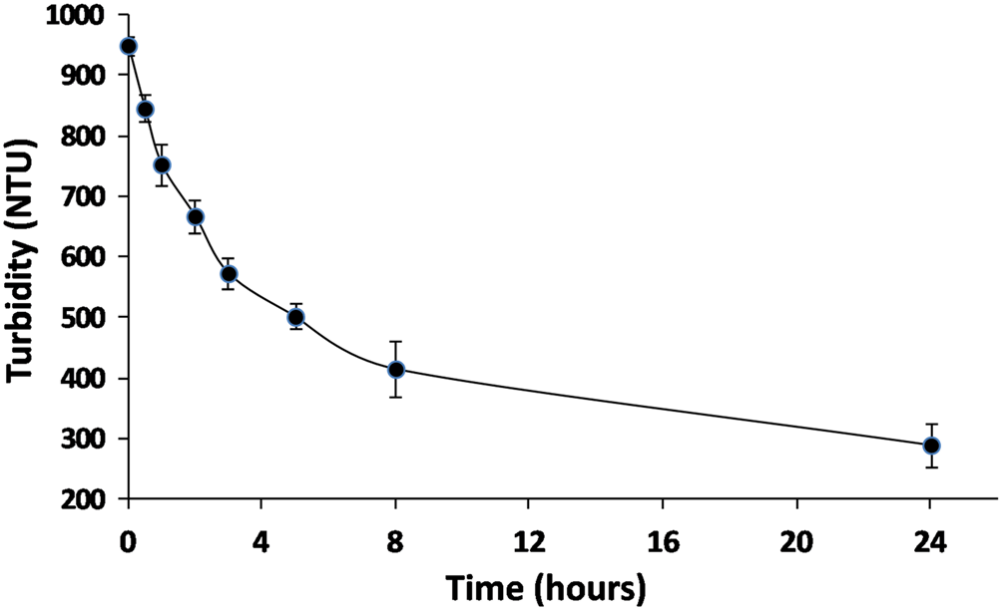
Sedimentation study (evaluated by turbidity measures) of a batch treatment of a red wine with a dose of 5 g/L of adsorbent material (bifunctionalized mesoporous xerogel).

In order to evaluate the behavior of the functionalized solid when used in the batch treatment of a wine, sedimentation time of the solid in a red wine was evaluated. Figure 2 shows the evolution of wine turbidity during the first 24 h. As can be seen, after addition and homogenization of the solid, turbidity decreased by only 30% during the first two hours (the time needed to reach extraction equilibrium according to previous results^19^) and, after 24 h, the percentage of turbidity due to the solid was around 30%. Separation of the remaining solid could be carried out using the usual filtration processes used in wineries. Therefore, the application protocol for batches can be similar to the use of bentonite.

Regarding reactivation and reutilization of the adsorbent materials after a first and a second use in a young red wine, the filtrated solid (reactivated by a washing process) was used for the retention of the 5 biogenic amines (histamine, cadaverine, putrescine, spermine and spermidine) in a synthetic wine media. The percentage removed of each amine from the synthetic media is presented in Supplementary Table S3. As can be seen, the adsorbent maintained very similar removal capacity to the original material after two uses. Therefore, by the application of a simple cleaning/reactivation treatment with acid solutions, the adsorbent can be recovered and re-used at least two more times. It is remarkable that the slight coloration observed for the re-used materials (after cleaning and reactivation; Supplementary Fig. S1) did not affect the removal properties.

## Conclusions

The mesoporous silica material bi-functionalized with phosphonic and sulfonic acids allowed the removal of histamine, putrescine, cadaverine, spermine and spermidine from wines, requiring adjustment of the dose according to the removal requirements and the initial levels in wines. Although a strong decrease in the content of several ethyl esters was observed just after treatments, after several days, the content was re-equilibrated and less important differences compared to the control wines were observed. At sensory level, the perception of these differences will depend on the complexity of the global aroma, so the proposed technique would be more adequate for wines aged in barrels than for young wines. The impact on wine color was less important but perceptible in most cases, probably due to a slight decrease in the pH of treated wines. At a practical level, the nature of the silica material proposed would allow its use in batches in a similar way to bentonite with adjustment of the protocol in order to recover and recycle the absorbent.

## Materials and methods

### Reagents

BA standards of histamine, putrescine, cadaverine, spermidine, spermine, 2-phenylethylamine, tyramine, isoamylamine and n-heptylamine (purity > 97%) were obtained from TCI Europe chemicals (Zwijndrecht, Belgium) and Across chemicals (Geel, Belgium). Tetraethyl orthosilicate (TEOS) (98%) from Alfa-Aesar (Kandel, Germany), 3-mercaptopropyltrimethoxysilane (MPTS) (95%) and (Diethylphosphatoethyl)triethoxysilane (DPETS) (95%) from ABCR (Karlsruhe, Germany), were used as precursors for the synthesis of functionalized silica. Bis[3-(silsequioxy)propyl] tetrasulfide was kindly donated by Sikemia (Montpellier, France). Triblock copolymer Pluronic P123 (Poly(ethylene glycol)-*block*-poly(propylene glycol)-*block*-poly(ethylene glycol)) from Aldrich (St. Louis, USA), and polyethylenglycol (PEG) 20,000 from Alfa-Aesar, were used as surfactants for the synthesis of silica by sol–gel route. Hydrochloric acid (37%), ethanol (> 99.8%), and nitric acid (65%) and dichloromethane (> 99.8%) from VWR (Fontenay sous Bois, France), and sulfuric acid (98%), Triphenylphosphine (99%), dioxane (99.8%), meta-chloroperoxybenzoic acid (99%) and diethylethoxymethylenemalonate (DEEMM), from Sigma-Aldrich (Steinheim, Germany), and ammonia (30%) from Carlo-Erba (Val de Reuil, France), were obtained.

### Synthesis of adsorbent materials

Lamellar, mesoporous xerogel (SBA-15 type) and macroporous xerogel bifunctionalized (with phoshonic and sulfonic acids) materials were synthesized according to Rodríguez-Bencomo & Mehdi^19^. For xerogel materials, a precursor molar ratio of 8:1:1 of TEOS, DPETS and MPTS was used. For SBA type material, precursors were added to a mixture of H_2_O, HCl and P123 in a molar ratio of 1:140:13.3:0.015. The mixture was heated and stirred at 40 °C for 20 h. The aging process was carried out at 60 °C for 24 h. After washing with water, surfactant was removed by soxhlet extraction with ethanol. For the macroporous xerogel material, the precursor mixture was added to a mixture (previously stirred 30 min in an ice bath) of H_2_O, HNO_3_ and PEG in a molar ratio of 1:13.4:0.26:0.0013. The mixture was cooled in an ice bath and stirred for 30 min and was then aged for 72 h at 40 °C. The solid was washed with water and was then treated with ammonium solution 0.1 M for 24 h at 40 °C. Surfactant was removed by soxhlet extraction with ethanol. Thiol group of MPTS were transformed into sulfonic acid by treatment with H_2_O_2_ (33%) and H_2_SO_4_ (2 M). Diethylphosphonate groups were transformed into phosphonic acid by treatment with HCl (37%). Finally, solids were washed with water.

For the synthesis of the lamellar sulfonic acid-functionalized material, Bis[3-(silsequioxy))propyl]-tetrasulfide was treated with triphenylphosphine and water (molar ratio 1:6.5:8.1) in acidic solution of dioxane (80 mL of dioxane with 10 drops of HCl (37%)). The mixture was stirred for 3 h at 40 °C under argon. The product was washed with dioxane and acetone and was then treated with 2 equivalents of meta-chloroperoxybenzoic acid in dichloromethane for 12 h at room temperature. The material obtained was washed with dichloromethane and dried under vacuum.

The degree of cation exchange functionalization of each material was evaluated by potentiometric titration (pH meter-CrisonBasic 20) with NaOH (0.2 N). The mill-equivalents of H^ +^ per gram of material were 1.62, 1.00 and 2.01 for the mesoporous, macroporous and lamellar materials, respectively.

### Wine samples and treatment of wines with the adsorbent materials

Wine samples were obtained from a retail shop. For the evaluation of effectiveness of the treatments with the adsorbent materials, BA of wines were analyzed and their concentration in wine was adjusted by addition of pure BA standard solutions up to the following levels: histamine (7.00 mg/L), putrescine (2.00 mg/L), cadaverine (1.50 mg/L), spermine (1.50 mg/L), spermidine (1.50 mg/L), 2-phenylethylamine (2.00 mg/L), isoamylamine (1.00 mg/L) and tyramine (7.00 mg/L). In accordance with previous results^19^, the contact time of the adsorbents with the wines was fixed at 3 h, with continuous stirring. The dose of adsorbent materials used in the experiments was 5 g/L, except when experimenting with the effect of the dose (doses ranged from 1 to 5 g/L). A 40 mL wine volume was treated in caped amber flasks of 50 mL and the headspace was flushed with nitrogen (or argon) to avoid oxidation phenomena during extraction. After treatment time, samples were filtered (0.2 µm). Wines treated and untreated were stored at 4 °C until analysis. All experiments were carried out in duplicate.

### Analysis of BA in wines

BA in wine samples were derivatized with DEEMM in order to obtain the aminoenone derivatives according to the method published by Gómez-Alonso et al.^34^ In a 5 ml screw-cap vial, 1.75 ml of borate buffer 1 M (pH 9), 1 ml of wine sample, 50 µL of internal standard solution (n-heptylamine at 50 mg/L), 30 µL of DEEMM and 0.75 mL of methanol, were added. The derivatization reaction was carried out in an ultrasonic bath for 30 min. Excess DEEMM were degraded at 70 °C for 2 h. After that, samples were filtered with 0.22 µm, diluted 1:1 with a dilution solvent (methanol, synthetic wine and phosphate buffer in the same ratio as the derivatization reaction), and 1 µL was injected in the LC system.

Chromatographic analysis was performed using an Agilent 1260 infinity instrument (nano-LC system) equipped with a Chip/MS interface (Chip cube), and an Agilent 6460 triple quadrupole mass spectrometer (Agilent Technologies, Waldbronn, Germany). Chromatographic separation was performed with an Agilent HPLC-polymeric chip that integrates a 40 nL enrichment column (4 mm) and a reverse phase column (43 mm × 75 µm) packed with 5 µm ZORBAX 300 SB-C18 particles. The mobile phases were (A) water—1 mM ammonium acetate, and 0.1% in formic acid and adjusted to pH 3.2 with NaOH; and (B) acetonitrile. For the analysis, the sample was loaded into the enrichment column with 98% of A at 2 µL/min. After that, analytes were separated in the analytical column (0.4 µL/min) using the following gradient: from 0 to 10 min 20–90% of B, then 90% B for a further 10 min, equilibrating the column under the initial conditions for 5 min. The Multiple Reaction Monitoring (MRM) parameters were optimized (under positive ionization conditions (ESI +)) for all analytes and the internal standard and are presented in Supplementary Table S4. Linearity of the calibration curves was tested for each compound in the different matrices considered (synthetic, white and red wines). Results were expressed in percentage of amine removed compared to the control sample.

### Wine analysis

Commercial wine samples were characterized for each experiment. Protein content was determined by the Bradford method and total content of peptides by the Doi’s method^35,36^. pH was measured with a pH meter (CrisonBasic 20). CIELab parameters (Color intensity and tonality) were calculated using MSCV software (https://www.unirioja.es/color/descargas.shtml) (illuminant D65, observer placed at 10°), the estimation of color change using CIELab parameters ((∆E_ab_) = ((∆L)^2^ + (∆a)^2^ + (∆b)^2^))^1/2^)^33^, and total polyphenol index (TPI) was determined according to OIV methods. All spectrophotometric measurements were performed with a Shimadzu UV-2450/2550 spectrophotometer.

Extraction of the volatile compounds from wines was carried out just after the treatment with solids by dispersive liquid–liquid extraction with dichloromethane/acetone and analysed by gas chromatography-mass spectrometry (GC–MS). Briefly, 5 ml of wine were extracted with 870 µL of acetone and 225 µL of dichloromethane. A 30 µL of internal standards solution (100 mg/L of d5-ethyl butanoate, d5-ethyl hexanoate, d5-ethyl octanoate, d5-ethyl decanoate, d7-butyric acid and d4-phenylethanol) were added before extraction. The sample was vortexed for 5 min. The organic phase was recovered and directly injected in the chromatograph system (Thermo-Scientific GC/MS ISQ system). Quantifications were carried out by using calibration curves built with synthetic samples spiked with the analytes. Results for treated samples were expressed as a deviation percentage in relation to the levels in control samples. Analyses were carried out in duplicate.

After stabilization time (1 week), extraction of the linear ethyl esters (ethyl hexanoate, decanoate and octanote) was carried out by liquid–liquid extraction with dichloromethane and the extract was analysed by gas chromatography-flame ionization detection (GC-FID)^37^. Quantifications were performed by using calibration curves built with synthetic samples spiked with the analytes. All analyses were carried out in duplicate. Odor activity values of ethyl esters (OAV = Concentration/Odor threshold) were calculated by using the odor threshold obtained from the bibliography^38^.

Polyphenol compounds analysis (hidroxybenzoic acid derivatives, hidroxycinnamic acid derivatives and flavonoids) was carried out by direct injection of wine samples in a UPLC-TQMS system according to the method published in Vrhovsek et al.^39^.

### Sensory analysis

In order to evaluate the effect on aroma, 2 × 125 mL of 8 different red wines (2 young wines and 6 aged in barrels; monovarietals made using 6 different grape varieties) were treated with a dose of 5 g/L of the mesoporous adsorbent material (for 3 h). The extraction was carried out in 125 mL-caped amber flasks, the headspace was flushed with nitrogen and the sample was centrifugated and filtered after treatment. After 1 week of stabilization time, wines were evaluated by using triangle tests (treated wines vs. control wines). A panel of 15 students in the 4th year of the Enology degree at Rovira i Virgili University, with experience in wine tasting, evaluated the wines in a single session, in duplicate, in covered glasses at the tasting room of Rovira i Virgili University. Panelists were asked to respond only to differences in aroma in all triangle tests. The project had the consent of the Vice-Chancellor for Research at the Rovira i Virgili University to carry out the sensory analysis of the wines, involving human subjects. Rovira i Virgili University Institutional Review Board grants an exempt status for this type sensory analysis. All experiments were performed in accordance with the relevant guidelines and regulations for sensory analysis research with human participants according to the guidelines of the Institute of Food Science and Technology (https://www.ifst.org/our-resources/ifst-guidelines-ethical-and-professional-practices-sensory-analysis-foods). The privacy rights of human subjects were always observed and an informed consent was obtained from all participants. Color parameters, pH and linear ethyl esters contents of these wines were evaluated according to the methods described in the Wine analysis section.

### Evaluation of practical aspects for application in batches.

Sedimentation kinetics were evaluated in a glass tube containing 1 L of a red wine and a dose of 5 g/L of the mesoporous xerogel material. After total dispersion of the solid, the turbidity of the wine was measured for 24 h. Turbidity was measured by nephelometry (Turbiquant 1000 IR turbidimeter) (expressed in nephelometric turbidity units (NTU)).

Material cleaning and reactivation experiments for its re-use were carried out after treatment of a red wine with a dose of 5 g/L (two re-uses). The recovered material (by filtration) was cleaned twice with a 3 M HCl solution and washed with water until complete removal of HCl. After drying, the effectiveness of the material for the BA removal was evaluated on a synthetic wine fortified with the BA.

### Statistical analysis

One way-ANOVA, Two-way ANOVA, least significance difference (LSD) test and the Dunnett test were used to evaluate compositional differences between samples. STATISTICA program for Windows version 7.1 was used for data processing (StatSoft, Inc., 2005, www. statsoft.com).

## Supplementary information

Supplementary Information.
