# Supplementary material for: Removal of biogenic amines from wines by chemisorption on functionalized silica and effects on other wine components
Source: Sci Rep. 2020 Oct 14;10:17279. doi: 10.1038/s41598-020-74287-3 (PMC7560601; doi:10.1038/s41598-020-74287-3)
Supplement: Supplementary file 1 — Supplementary Information. [file 41598_2020_74287_MOESM1_ESM.docx]

**Title:** Removal of biogenic amines from wines by chemisorption on functionalized silica and effects on other wine components

**Authors:** Juan José Rodríguez-Bencomo ^1,^*^,^**, Peggy Rigou ^2^, Fulvio Mattivi ^3^, Francisco López ^4^ and Ahmad Mehdi ^1^

^1^ ICGM, Univ. Montpellier, CNRS, ENSCM, Montpellier, France.

^2^ UMR 1083 Sciences Pour l’Œnologie, INRA, Montpellier SupAgro, Univ. Montpellier, Montpellier, France.

^3^ University of Trento, Department of Cellular, Computational and Integrative Biology – CIBIO, San Michele all’Adige, Italy.

^4^ Department d’Enginyeria Química, Facultat d’Enologia, Universitat Rovira i Virgili, Tarragona, Spain.

* Corresponding author email: [jrbencomo@gmail.com](mailto:jrbencomo@gmail.com);

** Current address: Agrotecnio - Centre for Food and Agriculture Research. Av. Rovira Roure 191, 25198 Lleida (Spain).

**Supplementary material description**

**Figure S1.** Appearance of the functionalized mesoporous xerogel material before (left) and after its use in a red wine, washed and reactivated (right).

**Table S1.** Chemical formula, molecular weight (MW) and molecule length of the biogenic amines studied.

**Table S2.** Phenolic compounds of control wines and treated wine samples (adsorbent type and dose: mesoporous xerogel at 5g/L).

**Table S3.** Percentage of BA removed (%) by functionalized silica materials for a new and used/reactivated material.

**Table S4.** MRM parameters for the analysis of BA in wines by nanoLC-MS/MS.

**Figure S1.** Appearance of the functionalized mesoporous xerogel material before (left) and after its use in a red wine, washed and reactivated (right).

| **Table S1. Chemical formula, molecular weight (MW) and molecule length of the biogenic amines studied**. | | | |
| --- | --- | --- | --- |
| ***Biogenic amine*** | ***MW (g/mol)*** | ***Molecule Length ^a^ (nm)*** | ***Formula*** |
| *Histamine* | 111.15 | 0.789 | 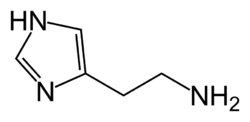 |
| *Putrescine* | 88.15 | 0.783 | 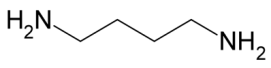 |
| *Cadaverine* | 102.18 | 0.777 | 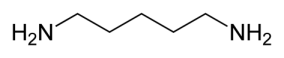 |
| *Spermidine* | 145.25 | 0.937 | 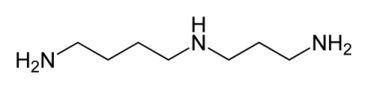 |
| *Spermine* | 202.34 | 1.062 | 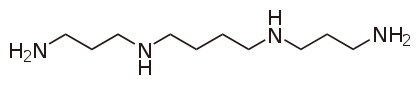 |
| *2-Phenylethylamine* | 121.18 | 0.812 | 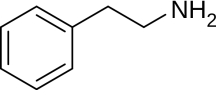 |
| *Tyramine* | 137.18 | 0.875 | 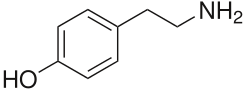 |
| *Isoamylamine* | 87.16 | 0.673 | 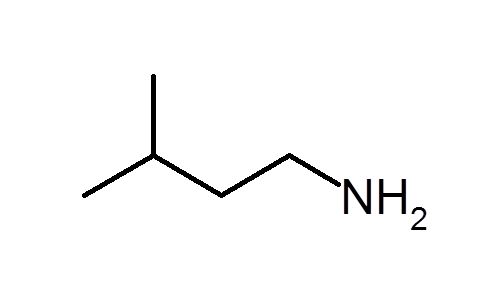 |
| ^a^ Obtained from http://molcalc.org/. | | | |

| **Table S2**. **Phenolic composition of control wines and treated wine samples (adsorbent type and dose: mesoporous xerogel at 5g/L).** | | | | | | |
| --- | --- | --- | --- | --- | --- | --- |
|  | **Control wine content (peak area x 10^-4^)^a^** | | | **Treated wines (% deviation respect control)** | | |
| **Compound** | **Lagrein** | **Tempranillo** | **Pinot Noir** | **Lagrein** | **Tempranillo** | **Pinot Noir** |
| *Caftaric acid* | 7.8 ± 0.2 b | 4.0 ± 0.1 a | 16.6 ± 0.5 c | -0.3 ± 2.7 | -15.1 ± 3.2 * | -6.9 ± 2.3 |
| *Fertaric acid* | 1.0 ± 0.0 a | 2.0 ± 0.1 b | 3.9 ± 0.1 c | 0.1 ± 3.5 | -20.8 ± 0.7 * | -13.2 ± 3.6 |
| *p-Coumaric acid* | 0.15 ± 0.00 a | 2.9 ± 0.2 b | 0.23 ± 0.03 a | -6.6 ± 7.8 | -16.6 ± 0.8 * | -8.6 ± 8.4 |
| *Caffeic acid* | 0.20 ± 0.02 a | 1.7 ± 0.0 c | 0.89 ± 0.01 b | -6.2 ± 0.8 | -6.6 ± 1.3 * | 6.2 ± 4.5 |
| *3,5-Dihydroxybenzoic acid* | 0.71 ± 0.07 a | 2.3 ± 0.0 b | 0.79 ± 0.02 a | 5.5 ± 2.4 | -3.6 ± 1.6 | -2.6 ± 2.4 |
| *Gallic acid* | 0.71 ± 0.02 a | 1.0 ± 0.0 c | 0.82 ± 0.04 b | -4.5 ± 0.9 | -6.1 ± 2.2 | 0.3 ± 1.6 |
| *trans-Coutaric acid* | 5.0 ± 0.10 a | 6.2 ± 0.2 b | 11.3 ± 0.0 c | -6.5 ± 5.9 | -13.6 ± 0.8 * | -0.7 ± 6.5 |
| *Ellagic acid* | 0.86 ± 0.08 a | 1.4 ± 0.1 b | 1.0 ± 0.1 a | -8.6 ± 5.6 | -1.2 ± 0.2 | -7.4 ± 2.4 |
| *Ferulic acid* | 0.10 ± 0.01 a | 0.37 ± 0.06 b | 0.41 ± 0.04 b | 7.9 ± 9.4 | -17.6 ± 6.8 | -10.7 ± 7.5 |
| *Quercetin-3-Glucoronide* | 13.4 ± 0.1 c | 7.4 ± 0.5 a | 10.6 ± 1.1 b | -4.3 ± 4.4 | -11.9 ± 4.8 | -7.1 ± 0.8 |
| *Quercetin-3-Glucoside* | 3.1 ± 0.2 c | d-n.q. a | 0.36 ± 0.05 b | -6.1 ± 6.7 | d-n.q. | 6.1 ± 0.3 |
| *Myricetin* | 4.2 ± 0.2 c | 2.4 ± 0.1 b | 0.64 ± 0.01 a | -38.2 ± 8.4 * | -32.6 ± 8.6 * | -15.6 ± 6.3 |
| *Laricitrin* | 0.37 ± 0.05 b | 0.14 ± 0.01 a | 0.08 ± 0.0 a | -29.0 ± 12.5 | -28.3 ± 5.3 * | 22.2 ± 4.2 * |
| *Rutin* | 52.6 ± 1.2 c | 8.5 ± 0.0 b | d-n.q. a | -19.4 ± 9.8 | -2.3 ± 11.0 | d-n.q. |
| *Catechin* | 0.80 ± 0.01 b | 0.34 ± 0.0 a | 2.9 ± 0.1 c | -1.3 ± 8.3 | -1.3 ± 9.0 | 4.0 ± 1.1 |
| *Epicatechin* | 2.1 ± 0.0 b | 0.55 ± 0.01 a | 3.9 ± 0.1 c | 1.2 ± 0.9 | 0.0 ± 7.2 | -0.6 ± 0.6 |
| *Gallocatechin* | 0.46 ± 0.0 a | 0.71 ± 0 c | 0.49 ± 0.00 b | 0.6 ± 10.3 | 5.1 ± 0.3 * | -10.7 ± 6.6 |
| *Epigallocatechin* | 0.17 ± 0.01 b | 0.08 ± 0 a | 0.07 ± 0.00 a | 5.0 ± 23.9 | 27.6 ± 6.3 * | 0.4 ± 15.8 |
| *Procyanidin B1* | 1.7 ± 0.0 a | 1.7 ± 0 a | 3.7 ± 0.1 b | 12.3 ± 7.6 | -2.3 ± 5.4 | -6.8 ± 1.1 |
| *Procyanidin B2* | 2.3 ± 0.0 b | 0.8 ± 0.04 a | 3.3 ± 0.1 c | 0.3 ± 7.0 | -5.2 ± 3.7 | -5.6 ± 2.3 |
| ^a^ Different letters indicate statistical differences in ANONA (p < 0.05) and LSD test.  *Indicate statistical differences in ANOVA (p<0.05) and in Dunnet test respect to the control wines (0% indicate the same concentration as the control wine). In addition, the criterion of a deviation higher than ±15% was applied to consider that the samples were different to produce an impact on wine characteristics.  d-n.q: Detected-not quantified. | | | | | | |

| **Table S3. Percentage of BA removed by functionalized silica material for a new and used/reactivated material.** | | | |
| --- | --- | --- | --- |
|  | **Adsorbent New** | **Adsorbent cycle 2** | **Adsorbent cycle 3** |
| *Histamine* | 98.4 ± 1.5 | 98.1 ± 0.1 | 98.3 ± 0.4 |
| *Cadaverine* | 98.9 ± 0.1 | 98.8 ± 0.1 | 98.8 ± 0.1 |
| *Putrescine* | 98.6 ± 0.1 | 98.6 ± 0.1 | 98.7 ± 0.1 |
| *Spermine* | 100 ± 0 | 100 ± 0 | 100 ± 0 |
| *Spermidine* | 100 ± 0 | 100 ± 0 | 97.5 ± 0.2 * |
| *Indicate statistical differences in ANOVA (p<0.05) and in Dunnet test respect to the adsorbent new. Removal values of 100 ± 0 indicate that the amine was not detected in the treated sample. | | | |

| **Table S4. MRM parameters for the analysis of BA in wines by nanoLC-MS/MS** | | | | |
| --- | --- | --- | --- | --- |
|  | **Precursor Ion**  **(m/z)** | **Product Ion (m/z)** | **Fragmentor** | **Collision Energy** |
| *Histamine* | 304,2 | 258,3 (Q) | 135 | 14 |
|  | 304,2 | 213.8 | 135 | 14 |
| *Putrescine* | 451,2 | 405,2 (Q) | 175 | 1 |
|  | 451,2 | 313.1 | 175 | 1 |
| *Cadaverine* | 465,2 | 419,1 (Q) | 55 | 2 |
|  | 465,2 | 373.2 | 55 | 2 |
| *Spermine* | 735,3 | 416,1 (Q) | 150 | 40 |
|  | 735,3 | 474.2 | 150 | 40 |
| *Spermidine* | 678,3 | 348,3 (Q) | 215 | 12 |
|  | 678,3 | 540.3 | 215 | 12 |
| *Tyramine* | 330,0 | 284,1 (Q) | 135 | 16 |
|  | 330,0 | 212.3 | 135 | 16 |
| *2-Phenylethylamine* | 314,1 | 268,2 (Q) | 195 | 10 |
|  | 314,1 | 195.9 | 195 | 10 |
| *Isoamylamine* | 280,1 | 162,3 (Q) | 135 | 24 |
|  | 280,1 | 234.1 | 135 | 24 |
| *n-heptylamine (IS)* | 308,2 | 262,3 (Q) | 135 | 22 |
|  | 308,2 | 190.1 | 135 | 22 |
| Q= indicate quantifier transition. IS= Internal Standard. Cell Accelerator was 4 for all transitions. | | | | |
